# Bromination of L-tryptophan in a Fermentative Process With *Corynebacterium glutamicum*

**DOI:** 10.3389/fbioe.2019.00219

**Published:** 2019-09-18

**Authors:** Kareen H. Veldmann, Steffen Dachwitz, Joe Max Risse, Jin-Ho Lee, Norbert Sewald, Volker F. Wendisch

**Affiliations:** ^1^Genetics of Prokaryotes, Faculty of Biology & Center for Biotechnology (CeBiTec), Bielefeld University, Bielefeld, Germany; ^2^Organic and Bioorganic Chemistry, Faculty of Chemistry & Center for Biotechnology (CeBiTec), Bielefeld University, Bielefeld, Germany; ^3^Fermentation Technology, Technical Faculty & Center for Biotechnology (CeBiTec), Bielefeld University, Bielefeld, Germany; ^4^Major in Food Science and Biotechnology, School of Food Biotechnology and Nutrition, BB21+, Kyungsung University, Busan, South Korea

**Keywords:** *Corynebacterium*, fermentation, halogenation, amino acids, 7-bromo-L-tryptophan

## Abstract

Brominated compounds such as 7-bromo-l-tryptophan (7-Br-Trp) occur in Nature. Many synthetic and natural brominated compounds have applications in the agriculture, food, and pharmaceutical industries, for example, the 20S-proteasome inhibitor TMC-95A that may be derived from 7-Br-Trp. Mild halogenation by cross-linked enzyme aggregates containing FAD-dependent halogenase, NADH-dependent flavin reductase, and alcohol dehydrogenase as well as by fermentation with recombinant *Corynebacterium glutamicum* expressing the genes for the FAD-dependent halogenase RebH and the NADH-dependent flavin reductase RebF from *Lechevalieria aerocolonigenes* have recently been developed as green alternatives to more hazardous chemical routes. In this study, the fermentative production of 7-Br-Trp was established. The fermentative process employs an l-tryptophan producing *C. glutamicum* strain expressing *rebH* and *rebF* from *L. aerocolonigenes* for halogenation and is based on glucose, ammonium and sodium bromide. *C. glutamicum* tolerated high sodium bromide concentrations, but its growth rate was reduced to half-maximal at 0.09 g L^−1^ 7-bromo-l-tryptophan. This may be, at least in part, due to inhibition of anthranilate phosphoribosyltransferase by 7-Br-Trp since anthranilate phosphoribosyltransferase activity in crude extracts was half-maximal at about 0.03 g L^−1^ 7-Br-Trp. Fermentative production of 7-Br-Trp by recombinant *C. glutamicum* was scaled up to a working volume of 2 L and operated in batch and fed-batch mode. The titers were increased from batch fermentation in CGXII minimal medium with 0.3 g L^−1^ 7-Br-Trp to fed-batch fermentation in HSG complex medium, where up to 1.2 g L^−1^ 7-Br-Trp were obtained. The product isolated from the culture broth was characterized by NMR and LC-MS and shown to be 7-Br-Trp.

## Introduction

Brominated tryptophan is typically not found in free form in Nature, but as a biosynthetic precursor in complex structures that for example occur in sponges and lower marine invertebrates (Bittner et al., 2007). The brominated molecules often exhibit pharmaceutical and biological activities. For example, TMC-95A which derives from 7-bromo-l-tryptophan (7-Br-Trp) is biologically active against the chymotrypsin-like, trypsin-like, and peptidyl-glutamyl-peptide-hydrolyzing activities of the 20S proteasome of eukaryotic cells (Koguchi et al., 2000). Protease inhibitors may be promising candidates for tumor and inflammation therapies (Adams, 2004; Vergnolle, 2016). Free unprotected halotryptophans including 7-Br-Trp and 7-chloro-l-tryptophan (7-Cl-Trp) can serve as substrates for Pd-catalyzed cross-coupling reactions (Willemse et al., 2017) for example in the Suzuki-Miyaura cross-coupling in order to attach an aryl, heteroaryl, or alkenyl substituent to the indole ring (Roy et al., 2008). For this reaction, 7-Br-Trp is preferred because it is more reactive than 7-Cl-Trp (Corr et al., 2017). In addition, 7-Br-Trp can also be used in other transition metal-catalyzed cross couplings such as the Mizoroki-Heck reaction (Gruß et al., 2019) giving fluorescent styryl-tryptophans or the Sonogashira cross-coupling reaction (Sonogashira, 2002) to generate compounds such as the new-to-nature bromo-cystargamide or to selectively modify bromo-tryptophan residues as a component of a tripeptide (Corr et al., 2017). 7-Cl-Trp is not useful for the Sonogashira cross coupling reaction since it is too unreactive (Corr et al., 2017). Furthermore, 7-Br-Trp can easily be converted to 7-bromoindole, which may give rise to many indole derivates including the MOM-protected 7-bromoisatin, which is the precursor of the antimitotic agent diazonamide A (Nicolaou et al., 2002; Wang et al., 2007; Bartoli et al., 2014). Halogenation of l-tryptophan (Trp) involves two enzymes of the *reb* operon of *Lechevalieria aerocolonigenes*, the FAD-dependent halogenase RebH and the NADH-dependent flavin reductase RebF required for NADH-dependent redox cofactor regeneration (Nishizawa et al., 2005). The halogenase RebH from *L. aerocolonigenes* chlorinates Trp to 7-Cl-Trp, the precursor of rebeccamycin. While this enzyme also accepts bromide, it prefers chloride over bromide (Yeh et al., 2005). Purified cross-linked enzyme aggregates comprising RebH, RebF, and an alcohol dehydrogenase to regenerate NADH by oxidation of isopropanol have successfully been applied to the enzymatic bromination of Trp at the gram-scale (Frese and Sewald, 2015; Schnepel and Sewald, 2017). Fermentative production of 7-Cl-Trp has recently been established using recombinant *Corynebacterium glutamicum* (Veldmann et al., 2019).

Fermentation processes with *C. glutamicum* that serves as a work horse for the biotechnological production of different amino acids are scalable and in the case of l-lysine and l-glutamate applied at the million-ton scale (Wendisch, 2019). Fermentative processes unlike chemical synthesis routes do not require environmentally hazardous compounds (e.g., elemental chlorine or bromine) or protecting/activating groups because of the high stereo-and regioselectivities of the enzymes involved. Biotransformations using purified enzymes may suffer from low stability and low activity (e.g., of halogenases), especially under non-native reaction conditions in the presence of high substrate concentrations (Latham et al., 2017). Fermentative processes start from sugars and the biocatalyst is (re)generated during growth. Fermentative processes are excellent for synthesis if export of the product out of the cell is efficient and neither substrates nor products nor intermediates inhibit cellular metabolism.

*C. glutamicum* typically shows higher tolerance to many substances including organic acids, furan, and phenolic inhibitors present in lignocellulose hydrolysates (Sakai et al., 2007). Adaptive laboratory evolution led to increased tolerance to methanol (Leßmeier and Wendisch, 2015) or lignocellulose derived inhibitors (Wang et al., 2018). Thus, *C. glutamicum* was engineered for production of carboxylic acids such as pyruvate (Wieschalka et al., 2012) and succinate (Litsanov et al., 2012), oxoacids such as 2-ketoisovalerate (Krause et al., 2010) and 2-ketoisocaproate (Bückle-Vallant et al., 2014), alcohols such as ethanol (Inui et al., 2004a), isobutanol (Blombach et al., 2011), and *n*-propanol (Siebert and Wendisch, 2015), polymers such as polyhydroxyalkanoate (Ma et al., 2018). As industrial amino acid producer *C. glutamicum* is ideal for fermentative production of various other nitrogenous compounds such as the cyclic amino acid pipecolic acid (Pérez-García et al., 2016), the ω-amino acids γ-aminobutyrate (Kim et al., 2013; Jorge et al., 2016; Pérez-García et al., 2016) and 5-aminovalerate (Rohles et al., 2016; Jorge et al., 2017), the diamines putrescine (Schneider and Wendisch, 2010) and cadaverine (Tateno et al., 2009; Kim et al., 2018) and alkylated and hydroxylated amino acids such as *N*-methylalanine (Mindt et al., 2018) and 5-hydroxy-isoleucine (Wendisch, 2019). Noteworthy, several excellent *C. glutamicum* producer strains have been developed for production of muconic acid (Becker et al., 2018), phenylpropanoids (Kallscheuer and Marienhagen, 2018), *para*-hydroxybenzoic acid (Purwanto et al., 2018), and protocatechuate (Wendisch et al., 2016; Lee and Wendisch, 2017).

**Figure 1:**
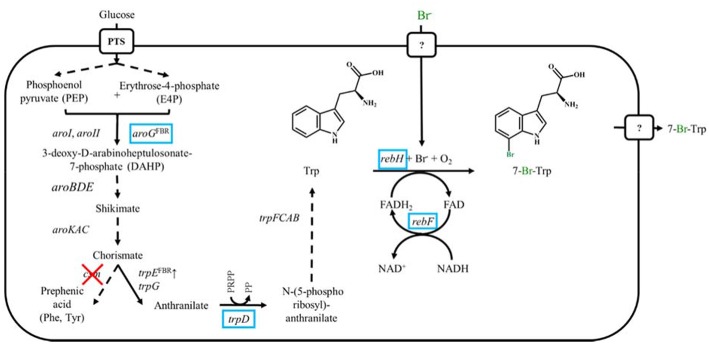
Schematic representation of metabolic engineered *C. glutamicum* overproducing Trp and 7-Br-Trp. Genes names are shown next to reaction represented by the arrows. Dashed arrows show several reactions. Heterologously expressed genes are marked by blue boxes, endogenously overexpressed genes are marked by ↑ and deleted genes are showed by red crosses. FBR, feedback resistant.

Accordingly, the Trp overproducing strain Tp679 (Purwanto et al., 2018) served as excellent base strain for halogenation of Trp (Veldmann et al., 2019). In *C. glutamicum* wildtype mutant *trpE* encoding a feedback resistant anthranilate synthase component 1 from *C. glutamicum* and *trpD* encoding an anthranilate phosphoribosyltransferase from *E. coli* were overexpressed to channel the flux from chorismate to Trp. The chorismate mutase *csm* was deleted to prevent the formation of the by-products l-phenylalanine and l-tyrosine. The precursor supply was optimized with the overexpression of *aroG* encoding a feedback resistant 3-deoxy-d-arabinoheptulosonate-7-phosphate synthase from *E. coli* (Figure 1). The production of 7-Cl-Trp had already been established with the Trp producing *C. glutamicum* strain overexpressing *rebH* and *rebF*. The strain produced about 0.1 g L^−1^ of 7-Cl-Trp (Veldmann et al., 2019). However, bromination of Trp *in vivo* has not yet been described as basis of a fermentative process leading 7-Br-Trp or other brominated tryptophans. Here, we describe the production of 7-Br-Trp with the above described Trp overproducing *C. glutamicum* strain expressing *rebH* and *rebF* in media with low chloride, but high bromide concentrations. The process was upscaled in bioreactors with a volume of 2 L and 7-Br-Trp was isolated and characterized by NMR and MS.

## Materials and Methods

### Bacterial Strains and Growth Conditions

Bacterial strains and plasmids used in this study are listed in Table 1. *Escherichia coli* DH5α (Hanahan, 1983) was used for cloning the plasmid constructs. *E. coli* and *C. glutamicum* were regularly grown in lysogeny broth medium (LB medium) in 500 mL baffled flasks at 120 rpm at 37°C or 30°C, respectively. For growth and production experiments *C. glutamicum* was inoculated in CGXII minimal medium (Eggeling and Bott, 2005) in 500 or 100 mL baffled flasks (filling volume 10%) to an optical density (OD_600_) of 1 and incubated at 120 rpm. Growth was monitored by measuring the optical density at 600 nm using a V-1200 spectrophotometer (VWR, Radnor, PA, USA). For toxicity test *C. glutamicum* was grown in the BioLector® (M2P Labs) in CGXII medium supplemented with the substance to be tested. To produce 7-Br-Trp, CGXII minimal medium or HSG rich medium (40.0 g L^−1^ glucose, 13.5 g L^−1^ soy peptone, 7 g L^−1^ yeast extract, 0.01 g L^−1^ NaCl, 2.3 g L^−1^ K_2_HPO_4_, 1.5 g L^−1^ KH_2_PO_4_, 0.249 g L^−1^ MgSO_4_ × H_2_O) were used and supplemented with 50 mM NaBr. Strains derived from Tp679 were supplemented additionally with 1.37 mM l-tyrosine and 1.5 mM l-phenylalanine in minimal medium. If necessary, the growth medium was supplemented with kanamycin (25 μg mL^−1^) and/or spectinomycin (100 μg mL^−1^). Isopropyl-β-d-1-thiogalactopyranoside (IPTG) (1 mM) was added to induce the gene expression from the vector pEKEx3 (Stansen et al., 2005).

**Table 1:** Strains and plasmids used in this work.

**Strains and plasmids**	**Description**	**Source**
**Strains**		
WT	*C. glutamicum* wild type, ATCC 13032	ATCC
Tp679 (pCES208-*trpD*)	Δ*csm* Δ*trpL*::P_*ilv*CM1_-*trpE*^FBR^ Δ*vdh*::P_*ilv*CM1_-*aroG*^FBR^ with pCES208-*trpD*	Purwanto et al., 2018
HalT2	Tp679 (pCES208-*trpD*)(pEKEx3-optimRBS-*rebH*-*rebF*)	Veldmann et al., 2019
**Plasmids**		
(pCES208-*trpD*)	Kan^R^, pCES208 overexpressing *trpD* from *E. coli* with P_ilvCM1_	Purwanto et al., 2018
(pEKEx3-optimRBS-*rebH*-*rebF*)	Spec^R^, pEKEx3 overexpressing *rebH, rebF* from *L. aerocolonigenes* with optimized RBS for *rebH*	Veldmann et al., 2019

### Determination of the Specific Activity of the Anthranilate Phosphoribosyltransferase TrpD

The anthranilate phosphoribosyltransferase overproducing strain Tp679 (pCES208-*trpD*) was inoculated from an overnight culture and was cultivated for 24 h in LB medium at 30°C with 120 rpm before cells were centrifuged for 10 min at 4°C and 4,000 rpm and stored at −20°C. After resuspension in 100 mM Tricine buffer (pH 7.0), the cells were sonicated for 9 min at 55% amplitude and 0.5 cycles on ice in the UP200S Ultrasonic Processor from Hielscher Ultrasound Technology. The supernatant obtained after centrifugation (60 min, 4°C, 16,400 rpm) was used as crude extract for the enzyme assay. The activity was assayed fluorometrically by monitoring the decrease of anthranilate (Ant) at room temperature. The reaction mixture with a final volume of 1 mL contained 100 mM Tricine buffer (pH 7.0), 15 μM Ant, 0.3 mM PRPP, 10 mM MgCl_2_, and the crude extract and was filled in a quartz glass cuvette (Hellma Analytics, High Precision cell, Light Path 10 × 4 mm). Ant was detected by fluorescence at 325 nm excitation and 400 nm emission wavelength with the Shimadzu Spectrofluorophotometer RF-5301PC. Protein concentrations were determined by the Bradford method (Bradford, 1976) with bovine serum albumin as reference. Means and errors from triplicates were calculated.

### Bioreactor Cultures Operated in Batch and Fed-Batch Mode

A 3.7 L KLF Bioengineering AG stirred tank reactor was used for the production of 7-Br-Trp. The fermentation was performed at pH 7.0, 30°C, and an aeration rate of 2 norm liter (NL) min^−1^. pH was controlled by automatic addition of phosphoric acid [10% (w/w)] and ammonium hydroxide [25% (w/w)]. Struktol®J647 (Schill and Seilenbacher, Boeblingen, Germany) serves as antifoam agent and was also added automatically. Samples were taken automatically every 2 h and cooled to 4°C until analysis.

For the batch fermentations the relative dissolved oxygen saturation (rDOS) of 15, 30, and 60%, respectively, was controlled by enhancing the stirrer speed gradually in steps of 2%. Two liter CGXII without MOPS but with 50 mM NaBr, 1.37 mM l-tyrosine, 1.5 mM l-phenylalanine, and 1 mM IPTG (added at timepoint 0 h) was used as culture medium.

For the fed-batch fermentation the initial volume was 2 L and a constant overpressure of 0.2 bar was adjusted. Due to the new findings (see **Figure 6**), the culture medium was changed to HSG rich medium supplemented with 50 mM NaBr and 1 mM IPTG (added at timepoint 0 h). The feeding medium contained 150 g L^−1^ ammonium sulfate, 400 g L^−1^ glucose, 5.14 g L^−1^ NaBr, 0.25 g L^−1^ l-tyrosine, and 0.25 g L^−1^ l-phenylalanine. Automatic control of the stirrer speed kept the rDOS at 30%. The feeding started automatically when rDOS exceeds 60% and stops when rDOS felt again under the set-point. Here, a pH of 7.0 was established and controlled by automatic addition of phosphoric acid [10% (w/w)] and potassium hydroxide (4 M). Instead of using ammonium hydroxide as alkali to avoid nitrogen limitation in batch cultures, potassium hydroxide was used in the fed-batch fermentation, since the HSG complex medium is nitrogen rich and, hence, a nitrogen limitation was excluded.

The titer and yield were calculated to the initial volume.

### Analytical Procedures

For the quantification of the extracellular Trp, 7-Br-Trp and anthranilate (Ant) a high-pressure liquid chromatography (HPLC) system was used (1200 series, Agilent Technologies Deutschland GmbH, Böblingen, Germany). The supernatants of the cell culture were collected by centrifugation (14,680 rpm, 20 min, RT) and further used for analysis. For detection of Ant, Trp, and the derivatives, samples were reacted with *ortho*-phthaldialdehyde (OPA) (Schneider and Wendisch, 2010). The amino acid separation was performed by a precolumn (LiChrospher 100 RP18 EC-5 μ (40 × 4 mm), CS-Chromatographie Service GmbH, Langerwehe, Germany) and a column (Li-Chrospher 100 RP18 EC-5 μ (125 × 4 mm), CS Chromatographie Service GmbH). The detection was carried out with a fluorescence detector (FLD G1321 A, 1200 series, Agilent Technologies) with the excitation and emission wavelengths of 230 and 450 nm, respectively. The quantification of carbohydrates and organic acids was done using a column for organic acids (300 × 8 mm, 10 mm particle size, 25 Å pore diameter, CS Chromatographie Service GmbH) and detected by a refractive index detector (RID G1362A, 1200 series, Agilent Technologies) and a diode array detector (DAD G1315B, 1200 series, Agilent Technologies) (Schneider et al., 2011).

### Analytical RP-HPLC and RP-HPLC-MS

Analytical HPLC was performed on a Shimadzu NexeraXR 20A System with autosampler, degasser, column oven, diode array detector, and a Phenomex Luna C18 column (2.9 μm, 50 × 2.1 mm) with a gradient (in 5.5 min from 5% B to 95% B, 0.5 min 95% B and back to 5% B in 3 min, total run time 9 min) at a flow rate of 650 μl/min and column oven temperature of 40°C. HPLC solvent A consists of 99.9% water and 0.1% TFA, solvent B of 99.9% acetonitrile and 0.1% TFA.

Analytical LC-MS was performed on an Agilent 6220 TOF-MS with a Dual ESI-source, 1200 HPLC system with autosampler, degasser, binary pump, column oven, diode array detector, and a Hypersil Gold C18 column (1.9 μm, 50 × 2.1 mm) with a gradient (in 11 min from 0% B to 98% B, back to 0% B in 0.5 min, total run time 15 min) at a flow rate of 300 μL/min and column oven temperature of 40°C. HPLC solvent A consisted of 94.9% water, 5% acetonitrile, and 0.1% formic acid, solvent B of 5% water, 94.9% acetonitrile and 0.1% formic acid. ESI mass spectra were recorded after sample injection via 1200 HPLC system in extended dynamic range mode equipped with a Dual-ESI source, operating with a spray voltage of 2.5 kV.

### NMR Spectroscopy

NMR spectra were recorded on a Bruker Avance III 500 HD (^1^H: 500 MHz, ^13^C: 126 MHz, ^19^F: 471 MHz). Chemical shifts δ [ppm] are reported relative to residual solvent signal (DMSO-*d*_6_, ^1^H: 2.50 ppm, ^13^C: 39.5 ppm). 2D spectra (COSY, HMQC, HMBC) spectra were used for signal assignment.

### High-Resolution MS

ESI mass spectra were recorded using an Agilent 6220 time-of-flight mass spectrometer (Agilent Technologies, Santa Clara, CA, USA) in extended dynamic range mode equipped with a Dual-ESI source, operating with a spray voltage of 2.5 kV. Nitrogen served both as the nebulizer gas and the dry gas. Nitrogen was generated by a nitrogen generator NGM 11. Samples are introduced with a 1200 HPLC system consisting of an autosampler, degasser, binary pump, column oven, and diode array detector (Agilent Technologies, Santa Clara, CA, USA) using a C18 Hypersil Gold column (length: 50 mm, diameter: 2.1 mm, particle size: 1.9 μm) with a short isocratic flow (60% B for 5 min) at a flow rate of 250 μL/min and column oven temperature of 40°C. HPLC solvent A consisted of 94.9% water, 5% acetonitrile, and 0.1% formic acid, solvent B of 5% water, 94.9% acetonitrile, and 0.1% formic acid. The mass axis was externally calibrated with ESI-L Tuning Mix (Agilent Technologies, Santa Clara, CA, USA) as calibration standard. The mass spectra were recorded in both profile and centroid mode with the MassHunter Workstation Acquisition B.04.00 software (Agilent Technologies, Santa Clara, CA, USA). MassHunter Qualitative Analysis B.07.00 software (Agilent Technologies, Santa Clara, CA, USA) was used for processing and averaging of several single spectra.

### Reversed-Phase Column Chromatography (GP1)

Automated column chromatography was performed on a Büchi Reveleris X2 with a binary pump and ELSD Detector using a Biotage Snap Ultra C18 column with a gradient (4 min at 5% B, up to 25% B in 14 min, in 1 min up to 100% B for 2 min and flushing with 80% B for 5 min, total run time 27 min) at a flow rate of 30 ml/min. Solvent A consisted of 99.9% water and 0.1% TFA, solvent B of 99.9% acetonitrile and 0.1% TFA.

### Isolation and Purification of 7-Bromo-l-tryptophan From HSG Rich Medium

7-Br-Trp was isolated from 30 ml HSG rich medium (3 × 10 ml) (see chapter growth conditions) by an automated reversed phase column chromatography. The crude medium was centrifuged (10,000 rpm, 4°C, 30 min) and filtrated over a short plug of celite. The crude filtrate was loaded on a 12 g C18-column and purified according to GP1. The TFA salt of 7-Br-Trp was isolated as a colorless solid (14.3 mg, 36 μmol). RP-column chromatography: *t*_R_ = 11.5 min; Anal. RP-HPLC: *t*_R_ = 3.3 min; LC-MS: *t*_R_ = 5.1 min; ^1^H NMR (500 MHz, DMSO-*d*_6_) δ [ppm] = 11.28 (d, ^3^*J* = 2.7 Hz, 1H, indole-N**H**), 8.16 (brs, 3H, N${\text{H}}_{3}^{+}$), 7.58 (d, ^3^*J* = 7.9 Hz, 1H, C4-**H**), 7.32 (d, ^3^*J* = 7.5 Hz, 1H, C6-**H**), 7.30 (d, ^3^*J* = 2.7 Hz, 1H, C2-**H**), 6.97 (dd, ^3^*J* = 7.8 Hz, ^3^*J* = 7.8 Hz, 1H, C5-**H**), 4.15 (dd, ^3^*J* = 7.1 Hz, ^3^*J* = 6.2 Hz, 1H, Cα-**H**), 3.27 (dd, ^2^*J* = 15.0 Hz, ^3^*J* = 5.7 Hz, 1H, Cβ-**H**), 3.22 (dd, ^2^*J* = 14.8 Hz, ^3^*J* = 6.9 Hz, 1H, Cβ-**H**); low res. MS (ESI): found [*m*/*z*] = 265.9 [M(^79^Br)-NH_2_]^+^, 267.9 [M(^81^Br)-NH_2_]^+^, 283.0 [M(^79^Br)+H]^+^, 285.0 [M(^81^Br)+H]^+^; calcd. [*m*/*z*] = 265.9 [M(^79^Br)-NH_2_]^+^, 267.9 [M(^81^Br)-NH_2_]^+^, 283.0 [M(^79^Br)+H]^+^, 285.0 [M(^81^Br)+H]^+^.

## Results

### Production of 7-Bromo-l-tryptophan in Flasks Culture

**Figure 2:**
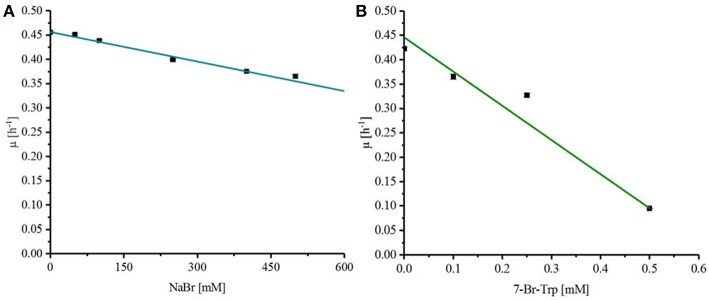
Response of *C. glutamicum* wild type to externally added NaBr **(A)** and 7-Br-Trp **(B)**. To determine the *K*_i_ for NaBr and 7-Br-Trp *C. glutamicum* wildtype was grown in CGXII minimal medium with 40 g L^−1^ glucose and different concentration of the substances to be tested. A linear regression was done to determine the half maximal specific growth rate with the substances to be tested. **(A)** NaBr concentrations between 0 and 500 mM were tested with *C. glutamicum*. **(B)** 7-Br-Trp concentrations between 0 and 0.5 mM were tested.

Fermentative processes are ideal if substrates, intermediates, and products do not inhibit growth and production. The effect of the substrate NaBr and the product 7-Br-Trp on growth of *C. glutamicum* was assessed when various concentrations of these compounds were added upon inoculation of *C. glutamicum* wild type to CGXII minimal medium with 40 g L^−1^ glucose. NaBr concentrations (0–500 mM) had a negligible effect on growth and it was estimated by extrapolation that the growth rate would be reduced to 50% at about 1.2 M NaBr (Figure 2A). Therefore, the use of NaBr as a substrate was presumed to be possible. By contrast, already low concentrations of the target product 7-Br-Trp inhibited growth in the BioLector® (M2P Labs). The half maximal specific growth rate of *C. glutamicum* was reached already at a concentration of about 0.32 mM or 0.091 g L^−1^ 7-Br-Trp (Figure 2B). This inhibition is threefold lower than previously observed with 7-Cl-Trp (*K*_i_ of about 0.1 mM or 0.024 g L^−1^ (Veldmann et al., 2019). We hypothesize that the difference is due to the hydration shell of chlorine substituents being smaller than that of a bromo substituent. Accordingly, we speculate that due to its smaller size 7-Cl-Trp can enter catalytic active centers and/or allosteric sites of enzymes easier than 7-Br-Trp and, thus, inhibitory effects are expected to be more pronounced. This may explain why the inhibitory effect of 7-Cl-Trp exceeds that of 7-Br-Trp.

**Figure 3:**
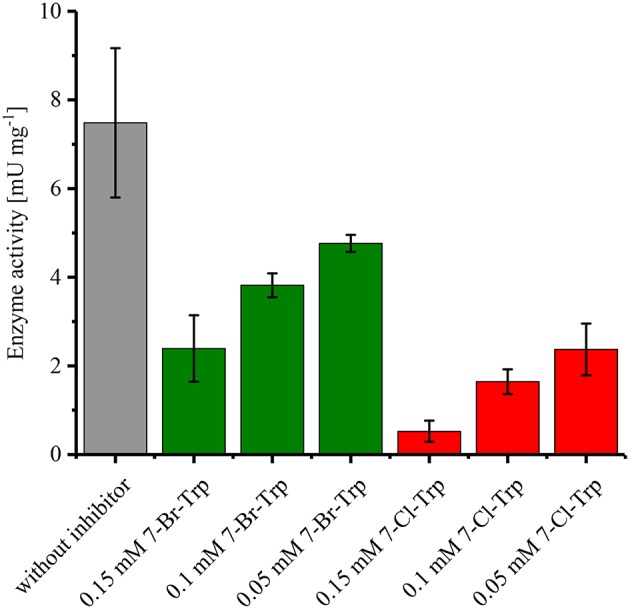
Specific activities of anthranilate phosphoribosyltransferase TrpD in the presence or absence of either 7-Br-Trp or 7-Cl-Trp. Crude extracts of *C. glutamicum* Tp679 (pCES208-*trpD*) grown in LB rich media were assayed for TrpD activity in the presence of different concentrations of either 7-Br-Trp or 7-Cl-Trp. Means and standard deviations of triplicates are shown.

Since it is known that halogenated Ant competitively inhibits Ant converting anthranilate phosphoribosyltransferase TrpD (Lesic et al., 2007), it was tested whether 7-Br-Trp inhibits anthranilate phosphoribosyltransferase in crude extracts of *C. glutamicum* Tp679 (pCES208-*trpD*). This strain possesses endogenous *trpD* on its chromosome and expresses *E. coli trpD* from a plasmid. Crude extracts of *C. glutamicum* Tp679 (pCES208-*trpD*) grown in LB rich medium were assayed for TrpD activity in the presence of different concentrations of either 7-Br-Trp or 7-Cl-Trp (Figure 3). The specific activity of anthranilate phosphoribosyltransferase was reduced to about one third by either 0.15 mM 7-Br-Trp or by 0.05 mM 7-Cl-Trp (Figure 3). Thus, inhibition by 7-Cl-Trp was more pronounced than inhibition by 7-Br-Trp, which showed a *K*_i_ value of about 0.03 g L^−1^. At least in part, the growth inhibition by 7-Br-Trp (Figure 2B) may be due to inhibition of anthranilate phosphoribosyltransferase by 7-Br-Trp (Figure 3). Since 7-Br-Trp exerts a lower inhibitory effect than 7-Cl-Trp and since the latter could be produced to a titer of 0.108 g L^−1^, i.e., five times as high as *K*_i_ (Veldmann et al., 2019), it is expected that *C. glutamicum* likely produces 7-Br-Trp only to relatively low concentrations as well.

**Figure 4:**
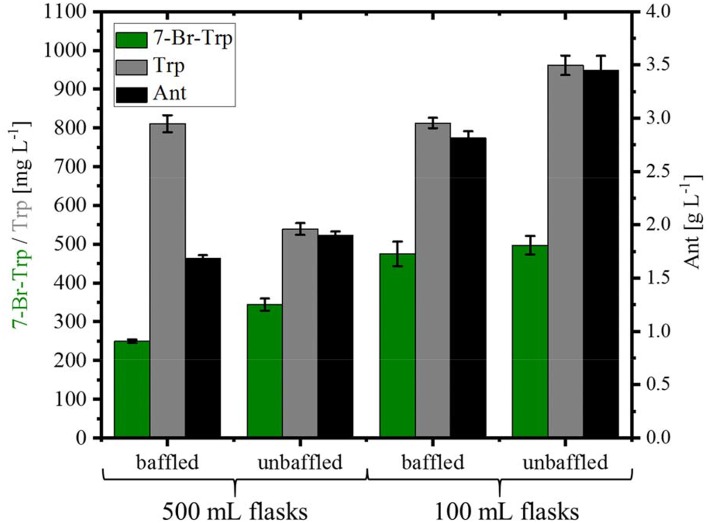
Production of 7-Br-Trp, Trp, and Ant by *C. glutamicum* HalT2 under different shake flask conditions. HalT2 was grown in CGXII with 40 g L^−1^ glucose after 72 h were measured the titers of 7-Br-Trp, Trp, and Ant. The filling volume was 10% of the flask volume. Means and standard deviations of three replicate cultivations are shown.

For the fermentative production of 7-Br-Trp, the *C. glutamicum* strain HalT2 was used. This strain was derived from the Trp overproducing strain Tp679 (pCES208-*trpD*), which overexpresses additionally genes encoding FAD-dependent halogenase RebH and NADH-dependent flavin reductase RebF from the expression vector pEKEx3 (Veldmann et al., 2019). In our previous study, we tried to optimize RebF and RebH gene expression. On the one hand, a more active promoter helped increase RebH and RebF activities, on the other hand, production could be improved as consequence of optimizing the ribosome binding site [and thus, translation initiation efficiency; (Veldmann et al., 2019)]. Bioinformatics analysis revealed that the codon usage of RebH fits to the codon usage of *C. glutamicum* and hence was not further optimized. For RebF the codon usage fits to *C. glutamicum* except one triplet. The ribosome binding site was not optimized for RebF. HalT2 was inoculated in CGXII minimal medium with 40 g L^−1^ glucose and 50 mM NaBr in 500 mL baffled flasks (50 mL culture) to an OD_600_ of 1. At inoculation, 1 mM IPTG was added. The culture showed a specific growth rate 0.12 ± 0.01 h^−1^. After 72 h 0.25 ± 0.01 g L^−1^ 7-Br-Trp, 0.81 ± 0.02 g L^−1^ Trp and 1.69 ± 0.03 g L^−1^ Ant were measured (Figure 4). When the same strain was inoculated in 500 mL without baffles the specific growth rate was 0.11 ± 0.01 h^−1^ and the production of 7-Br-Trp increased by 38% to a titer of 0.34 ± 0.02 g L^−1^. Production of Trp was decreased by 34% to 0.54 ± 0.02 g L^−1^, but production of Ant increased to 1.91 ± 0.03 g L^−1^. Using 100 mL flasks with 10 mL culture was beneficial for production of 7-Br-Trp by *C. glutamicum* HalT2 as in baffled flasks 0.48 ± 0.03 g L^−1^ 7-Br-Trp were produced after 72 h and 0.81 ± 0.01 g L^−1^ Trp and 2.82 ± 0.06 g L^−1^ Ant accumulated. In 100 mL flasks without baffles 0.49 ± 0.02 g L^−1^ 7-Br-Trp were produced after 72 h and 0.96 ± 0.03 g L^−1^ Trp and 3.45 ± 0.13 g L^−1^ Ant accumulated (Figure 4). With the assumption that the oxygen supply is lower in the 100 ml than in the 500 ml flasks, the production was increased with less oxygen supply. The specific growth rate was lower in 100 mL flasks with baffles (0.09 ± 0.01 h^−1^ as compared to 0.13 ± 0.01 h^−1^). These results were unexpected since oxygen supply in 500 mL baffled flasks is considered higher than in 100 mL unbaffled flasks we expected higher 7-Br-Trp in 500 ml baffled flasks. Halogenase RebH requires FADH_2_ as cofactor, l-Trp, molecular oxygen and a halide salt as substrates. RebH regioselectively chlorinates or brominates l-Trp at the 7-position. FADH_2_ is regenerated by RebF, which reduces FAD to FADH_2_ in an NADH-dependent manner. NADH is provided by cellular metabolism (oxidation of glucose). RebH and RebF derive from the host organism *Lechevalieria aerocolonigenes* which has a growth optimum at 28°C (Parte, 2012), which fits well with the optimal growth temperature of *C. glutamicum* of 30°C. Nonetheless, the highest 7-Br-Trp titer observed (about 0.49 g L^−1^; Figure 4) exceeded the *K*_i_ value (about 0.09 g L^−1^; Figure 2B) about five-fold.

### Batch Production of 7-Bromo-l-tryptophan in a Bioreactor

**Figure 5:**
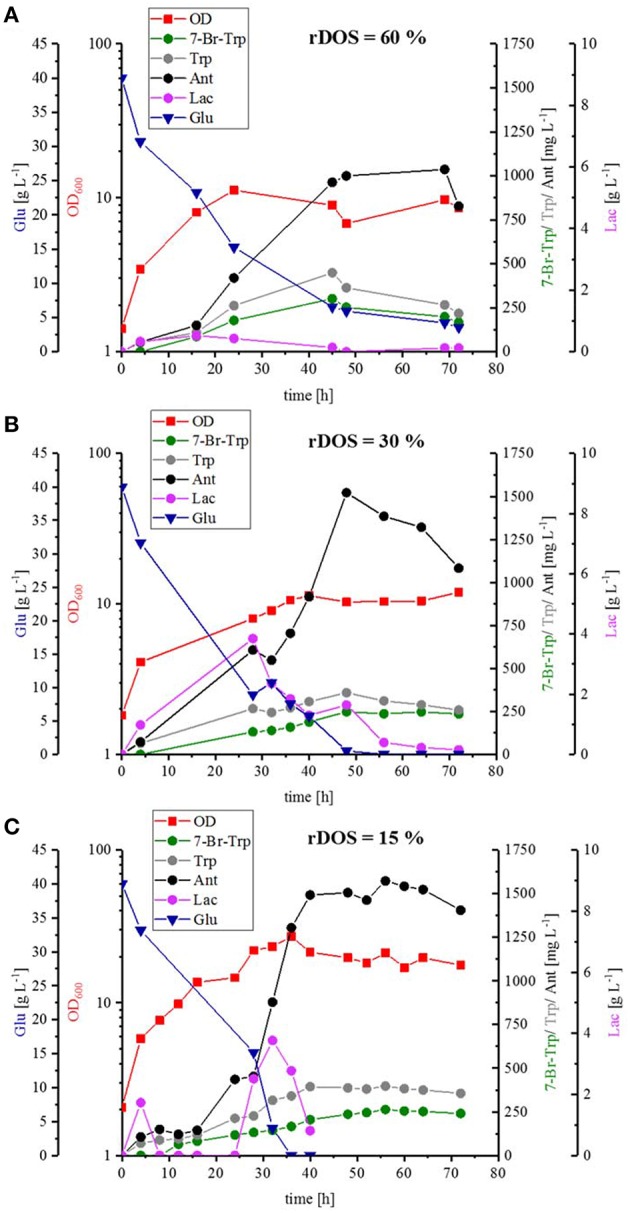
Batch fermentation of 7-Br-Trp by *C. glutamicum* HalT2 with three different rDOS. The data given include the glucose consumption [blue triangle], the OD_600_ [red squares], the production of 7-Br-Trp [green circles], Trp [gray circles], Ant [black circles], and Lac [light purple circles]. The initial culture volume was 2 L. **(A)** Batch fermentation with a rDOS set-point of 60%. **(B)** Batch fermentation with a rDOS set-point of 30%. **(C)** Batch fermentation with a rDOS set-point of 15%.

To scale up the fermentation process and to test the influence of pH control, optimal stirring and controlled oxygen supply, strain HalT2 was cultivated in a 3.7 L baffled bioreactor with a working volume of 2 L with three different rDOSs (rDOS = 15, 30, and 60%). Whereas, the maximal specific growth rate was comparable and in a range between 0.07 and 0.08 h^−1^, *C. glutamicum* HalT2 grew to a higher biomass concentration at rDOS of 15% (OD_600_ of 27; Figure 5C) than with rDOS at either 30 or 60% (OD_600_ of 11 and 12, respectively; Figures 5A,B). Glucose was utilized completely (with the exception of 3.6 g L^−1^ glucose remaining in the rDOS 60% bioreactor condition). Lactate accumulated transiently peaking at 32 h, 28 and 16 h with maximal concentrations of 3.8, 3.8, and 0.5 g L^−1^ lactate for the bioreactors operated at 15, 30, and 60%, respectively (Figure 5). The byproducts Trp and Ant accumulated to higher concentrations than 7-Br-Trp. Maximal 7-Br-Trp titers increased slightly with decreasing rDOS, i.e., 0.26, 0.26, and 0.30 g L^−1^ 7-Br-Trp for the bioreactors operated at 15, 30, and 60%, respectively (Figure 5). The corresponding yields on glucose were 6.6, 6.6, and 7.5 mg g^−1^. The yields on biomass differed to a larger extent since higher biomass concentrations were observed at low rDOS. At 15% rDOS, for example, an OD_600_ 21 (corresponding to 7.4 gCDW L^−1^) and a 7-Br-Trp titer of 0.26 g L^−1^ were observed at 56 h, which is equivalent to a 7-Br-Trp yield on biomass of 36 mg (gCDW)^−1^. At 30% rDOS, the 7-Br-Trp yield on biomass was almost two-fold higher [74 mg (gCDW)^−1^] and it was almost three-fold higher at 60% rDOS [95 mg (gCDW)^−1^]. This may indicate that less cells are required for 7-Br-Trp production at high rDOS and/or that growth proceeds to higher biomass concentrations at low rDOS.

### Fed-Batch Production of 7-Bromo-l-tryptophan in a Bioreactor

**Figure 6:**
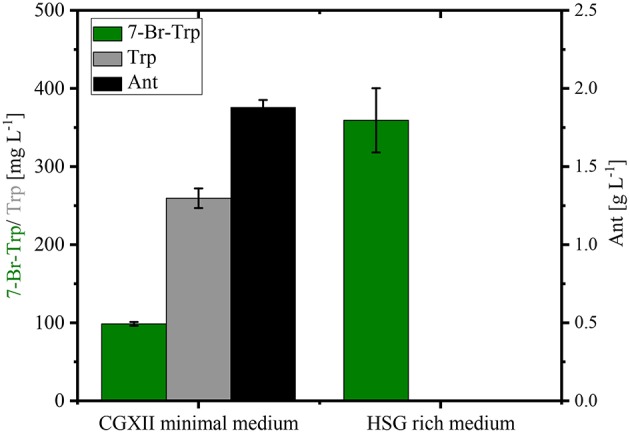
Production of 7-Br-Trp by *C. glutamicum* HalT2 in different media. HalT2 was grown in CGXII minimal medium and HSG rich medium with 40 g L^−1^ glucose. The strain was cultivated in 100 mL flasks with 10% filling volume. After 24 h were determined the production of 7-Br-Trp, Trp, and Ant. Means and standard deviations of three replicate cultivations are shown.

Stirred tank bioreactor cultivations operated in batch mode yielded lower titers (0.26–0.30 g L^−1^; Figure 5) than shake flask cultivation (up to about 0.49 g L^−1^; Figure 4). Under both conditions CGXII glucose minimal medium was used. Since fermentations are often performed in media containing complex sources such as yeast extract and/or protein hydrolysates, production of 7-Br-Trp by *C. glutamicum* HalT2 was compared in 100 mL baffled flasks with either CGXII glucose minimal medium or HSG rich medium containing yeast extract and soy peptone. Growth in the HSG rich medium was faster (a specific growth rate of 0.39 ± 0.02 h^−1^ as compared to 0.19 ± 0.01 h^−1^ in CGXII minimal medium). Within 24 h 0.36 ± 0.04 g L^−1^ 7-Br-Trp were produced in HSG rich medium, but only 0.10 ± 0.01 g L^−1^ 7-Br-Trp in the minimal medium (Figure 6). Moreover, the 7-Br-Trp yield on biomass in HSG rich medium [39 ± 5 mg g(CDW)^−1^] was higher than in CGXII minimal medium [6 ± 1 mg g(CDW)^−1^]. Notably, neither Trp nor Ant accumulated as byproducts in HSG rich medium, whereas 0.26 ± 0.01 g L^−1^ Trp and 1.88 ± 0.05 g L^−1^ Ant were produced in CGXII minimal medium (Figure 6).

**Figure 7:**
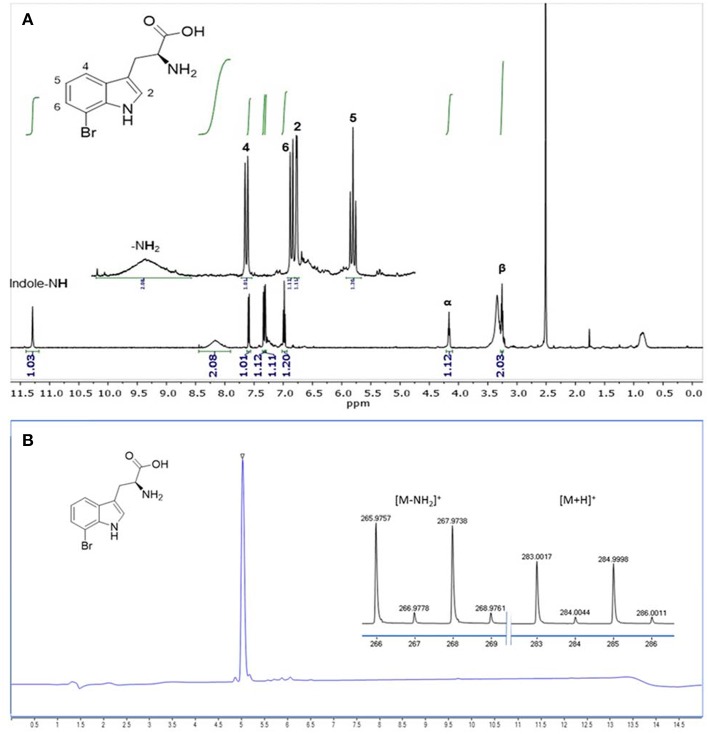
Analysis of 7-Br-Trp isolated from HSG medium by **(A)** ^1^H NMR (500 MHz, 298 K, DMSO-*d*_6_) and by **(B)** RP-HPLC-MS analysis. The ionization was performed with a Dual-ESI with a voltage of 2.5 kV leading to an expected deamination during the ionization process. The characteristic isotopic pattern of a single brominated species is clearly observable.

The product formed by *C. glutamicum* HalT2 in 30 ml HSG rich medium (3 × 10 ml) was isolated and purified in a single step by an automated reversed phase column chromatography. In total 14.3 mg (36 μmol) of 7-Br-Trp as a TFA salt were isolated. The product was identified as 7-Br-Trp by NMR studies. The purity (>95%) was verified by both NMR (Figure 7A) and LC-MS (Figure 7B) experiments.

**Figure 8:**
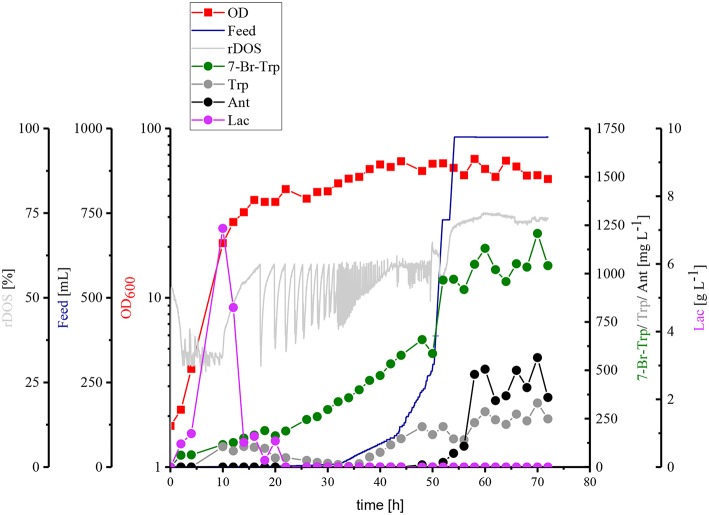
Fed-batch fermentation of 7-Br-Trp by *C. glutamicum* HalT2. The data given include rDOS [%] [light gray], the feed [blue], OD_600_ [red squares], 7-Br-Trp [green circles], Trp [gray circles], Ant [black circles], and Lac [light purple circles]. The initial volume was 2 L and 975 mL feed was added. The titer was calculated to the initial volume.

Taken together, HSG rich medium was chosen for fed-batch fermentation because the production occurred faster and the precursors Trp and Ant did not accumulate as byproducts and the specific growth rate in rich medium was two-fold higher than in CGXII medium as mentioned above (0.39 h^−1^ for HSG medium vs. 0.19 h^−1^ for CGXII medium). In Figure 6, the concentrations of Trp, Ant, and 7-Br-Trp after 24 h of cultivation in shake flasks are given. Most likely, in contrast to CGXII medium product formation was finished at this time point using rich medium. This was also confirmed by Figure 5, because 7-Br-Trp formation was not finished after 24 h of cultivation, irrespective of whether rDOS level was used. At 30% rDOS, the highest 7-Br-Trp yield on biomass of 74 mg (gCDW)^−1^ was reached as mentioned above. Therefore, the same set point was used for fed-batch fermentation. *C. glutamicum* HalT2 was used for the fed-batch fermentation to inoculate 2 L HSG rich medium containing 40 g L^−1^ glucose to an initial OD_600_ of 1.8 (Figure 8). The maximal specific growth rate was 0.32 h^−1^. A total feed of 975 mL was added in the whole process. Four major phases of the fed-batch fermentation could be distinguished. In the first (batch) phase *C. glutamicum* HalT2 grew to an OD_600_ of 37.3 within 18 h. Lactate accumulated transiently peaking at 7.0 g L^−1^ after 10 h. At 18 h, titers of 0.19 g L^−1^ 7-Br-Trp and 0.1 g L^−1^ Trp were observed. In the next phase (until 30 h when exponential feeding started), 7-Br-Trp was produced to a titer of 0.30 g L^−1^ with a yield on biomass of 0.7 mg (gCDW)^−1^ and a volumetric productivity of 10 mg L^−1^ h^−1^. Neither Trp nor Ant accumulated during this phase. The third phase is characterized by an exponential increase of the feed volume (at 50 h about 293 mL feed had been added and the OD_600_ reached 62), while the 7-Br-Trp concentration increased linearly to 0.66 g L^−1^. During this phase the volumetric productivity was 18 mg g^−1^ h^−1^ and the specific productivity was 1 mg gCDW^−1^ h^−1^. While Trp accumulated to a titer of 0.17 g L^−1^, Ant was not produced in the third phase. In the last phase that started at 50 h, the residual feed (682 mL) was added until 55 h. The 7-Br-Trp and Trp titers increased in parallel to 1.2 g L^−1^ and about 0.25 g L^−1^. Only in this last phase, Ant accumulated with Ant titers fluctuating around 0.5 g L^−1^ from 57 h to 72 h (Figure 8).

## Discussion

Heterologous expression of the genes *rebH* for FAD-dependent halogenase and *rebF* for NADH-dependent flavin reductase from the *reb* cluster of *L. aerocolonigenes* to enable regioselective chlorination of Trp at the 7 position (Nishizawa et al., 2005) in a Trp overproducing *C. glutamicum* strain (Purwanto et al., 2018) provided the basis for the development of fermentative processes for chlorination (Veldmann et al., 2019) and bromination of Trp (this study). Production of 7-Br-Trp by the engineered *C. glutamicum* strain was possible in glucose minimal media supplemented with sodium bromide.

*C. glutamicum* belongs to the group of bacteria that require chloride for growth at high (sodium) salt concentrations since growth was inhibited in the presence of high concentrations of sodium sulfate and sodium gluconate, but not of sodium chloride (Roeßler et al., 2003). It was postulated that chloride may enhance excretion of cytotoxic sodium ions by salt-induced Na^+^/H^+^ antiporters and/or simultaneous export of these anions via the ClC-type sodium channels as observed for *E. coli* (Iyer et al., 2002). The finding reported here that *C. glutamicum* can withstand high sodium bromide concentrations (*K*_i_ of about 1.2 M; Figure 2A) indicates that bromide may substitute for chloride to sustain growth of *C. glutamicum* at high sodium salt concentrations.

The engineered *C. glutamicum* strain produced 7-Br-Trp to higher titers (1.2 g L^−1^, Figure 8) than 7-Cl-Trp [0.1 g L^−1^; (Veldmann et al., 2019)]. This was surprising since pure RebH prefers chlorination (*k*_cat_ of 1.4 min^−1^) over bromination (*k*_cat_ of 0.4 min^−1^) (Yeh et al., 2005). Unlike in enzyme catalysis with pure RebH (Yeh et al., 2005; Payne et al., 2013), chloride could not be completely replaced by bromide since chloride is required for growth of *C. glutamicum* (s. above). However, at a low chloride concentration in the growth medium a high bromide salt supply (277-fold excess) allowed for bromination by the engineered *C. glutamicum* strain *in vivo*. The purified product of this fermentative process was shown to be 7-Br-Trp without detectable contamination by 7-Cl-Trp (Figure 7). Brominated natural products and intermediates are found predominantly in marine environments as ocean water contains a relatively high bromide ion concentration (Gribble, 1996). Moreover, halogenases which prefer bromination are more abundant in marine habitats, those preferring chlorination are encountered more often in terrestrial habitats (Van Peè, 2001). Thus, future process improvement may make use of halogenases preferring bromination over chlorination such as BrvH from *Brevundimonas* BAL3 (Neubauer et al., 2018) or three halogenases from *Xanthomonas campestris* pv. campestris strain B10046 (Ismail et al., 2019).

FAD-dependent halogenases require molecular oxygen (Bitto et al., 2008). In the reaction catalyzed by RebH, FADH_2_ binds to the FAD binding pocket of the RebH and reacts with molecular oxygen to flavin hydroperoxide (Andorfer et al., 2016). Flavin peroxide in turn oxidizes the halide anion (X^−^, X = Cl, Br) to hypohalous acid (HOX), which is channeled to the active tryptophan binding pocket. The role of the conserved lysine residue K79 in giving a haloamine intermediate (Yeh et al., 2007) is still under debate (Flecks et al., 2008). The hypohalous acid effects the regioselective electrophilic aromatic substitution of Trp resulting in halogenation at the C7 position (Andorfer et al., 2016). Thus, the supply of molecular oxygen to RebH within the *C. glutamicum* cell may be a bottleneck for halogenation of Trp. Since, of course, *C. glutamicum* requires oxygen for respiration, the response to increased molecular oxygen supply during growth-coupled fermentative production of 7-Br-Trp may be complex. Production of 7-Br-Trp was found to be higher under low oxygen supply in shake flask (although these are relatively ill-defined) or comparable under controlled conditions in bioreactor fermentations with rDOS of 15, 30, and 60% (Figures 4, 5). For different microorganisms the Monod constants for oxygen are in a range between 3.0 · 10^−4^ mg L^−1^ and 0.1 mg L^−1^ (Longmuir, 1954) Therefore, cell growth was not notably affected by oxygen supply even at a rDOS level of 15%. Surprisingly, biomass formation was declined with increasing rDOS set points as shown by the courses of OD_600_ and glucose in Figure 5. While glucose was depleted after 35 h for rDOS of 15% and 48 h for rDOS of 30%, respectively, nearly 4 g L^−1^ glucose remained at the end of cultivation (73 h) at a rDOS level of 60% also indicating a reduced biomass formation. However, halogenation by RebH-RebF requires molecular oxygen. Obviously, a better oxygen supply improves RebH-RebF activity, which counteracts biomass formation (see also max. OD_600_ mit tiefer gesetzten 600 values as given in line 719–722). This conclusion is supported by lower specific growth rates during stirred tank reactor cultivation (line 719) in contrast to shake flask cultivation (line 649–653, 694), despite a poorer oxygen input in the latter case. In addition, halogenation by RebH-RebF requires both molecular oxygen and NADH at the same time. This is difficult to achieve in fast growing cells as NADH is oxidized by the respiratory chain using molecular oxygen to generate a trans-membrane pH gradient and subsequently ATP. Moreover, if the oxygen supply is too high, the flavin-hydroperoxide formed upon reaction of FADH_2_ with molecular oxygen is hydrolyzed to yield H_2_O_2_. This is commonly observed for flavin-dependent enzymes like monooxygenases and halogenases (“oxygen dilemma”; Ismail et al., 2019). Thus, all reactions in *C. glutamicum* requiring FADH_2_ [e.g., *p*-hydroxybenzoate hydroxylase (Kwon et al., 2007) or flavin-dependent thymidylate synthase; (Kan et al., 2010)] or containing this flavin bound to the enzyme [e.g., membrane-associated malate dehydrogenase (acceptor) (EC 1.1.99.16); (Molenaar et al., 1998)] may be compromised at high oxygen levels in the presence of flavin reductase RebF. Thus, an aeration protocol ensuring optimal supply of oxygen for growth on the one hand and for RebH catalyzed bromination on the other hand remains to be developed.

Another optimization step for the fermentative production of 7-Br-Trp, would be the reduction of by-products, like l-lactate. *C. glutamicum* produces l-lactate from pyruvate via the NAD-dependent l-lactate dehydrogenase (encoded by *ldhA*) (Inui et al., 2004b) and is able to utilize the l-lactate as carbon source via the lactate dehydrogenase (encoded by *lldD*) (Stansen et al., 2005). Transient l-lactate accumulation (formed by LdhA and subsequently utilize by LldD) is often observed when glucose uptake is higher than oxygen uptake. Once the glucose uptake rate ceases, l-lactate is re-utilized. Transcription of *ldhA* is regulated by transcriptional regulator SugR (Engels et al., 2008; Toyoda et al., 2009). Under oxygen limitation glucose uptake exceeds oxygen uptake and l-lactate is produced by LdhA to regenerate NAD^+^ (Engels et al., 2008). Accordingly, transient l-lactate was more pronounced with low (rDOS of 15%, 30%, and fed-batch) as compared to high oxygen supply (rDOS of 60%). We have discussed these facts along with a strategy to avoid transient lactate formation, i.e., by deletion of *ldhA* as has been shown before (Inui et al., 2004b).

Inhibition by halogenated Trp appeared to be the major bottleneck to achieve high product titers. Growth as well as anthranilate phosphoribosyltransferase activity in crude extracts from *C. glutamicum* Tp679 (pCES208*-trpD*) were inhibited by 7-Br-Trp and 7-Cl-Trp (Figure 3 and Veldmann et al., 2019). When comparing growth and 7-Br-Trp production in CGXII and HSG media, the latter of which is a complex medium and contains about 0.5 mM Trp (data not shown), the specific growth rate in rich medium was two-fold higher than in CGXII medium (0.39 h^−1^ for HSG medium vs. 0.19 h^−1^ for CGXII medium, s. results section). Thus, addition of Trp may alleviate the growth inhibition as consequence of TrpD inhibition.

Previously, O'Gara and Dunican (1995) have shown that purified anthranilate phosphoribosyltransferase TrpD from *C. glutamicum* is inhibited by Trp (*K*_i_ of 0.83 mM) and by 5-methyl-l-tryptophan (*K*_i_ of 0.32 mM). In this study, anthranilate phosphoribosyltransferase activity in crude extracts was shown to be inhibited by 7-Br-Trp (*K*_i_ of about 0.1 mM) and by 7-Cl-Trp (*K*_i_ of about 0.06 mM). It should be noted that besides endogenous *trpD* on the *C. glutamicum* chromosome *E. coli trpD* was expressed from a plasmid. TrpD from *E. coli* is insensitive to Trp in the absence of *E. coli* TrpE (Ito and Yanofsky, 1969). Feedback resistant TrpD has been isolated from *C. glutamicum*, which was isolated from a tyrosine and phenylalanine double auxotrophic strain due to its resistance to analogs of Trp, tyrosine, phenylalanine, and 5-methyl-l-tryptophan. Feedback resistant TrpD from this *C. glutamicum* strain was shown to confer resistance to 5-methyl-l-tryptophan and 6-fluoro-l-tryptophan on *E. coli* (Herry and Dunican, 1993). Resistance to Trp derivatives with modifications at the 7 position of Trp such as 7-Br-Trp or 7-Cl-Trp has not been determined, thus, it cannot be inferred that the feedback resistant TrpD from *C. glutamicum* ATCC 21850 would alleviate the inhibition of *C. glutamicum* growth and/or TrpD activity by 7-Br-Trp and 7-Cl-Trp. Likely, other TrpD variants either from *C. glutamicum* or from *E. coli* have to be isolated after mutation and screening or by rational enzyme engineering. Alternatively, process intensification may involve fermentation strategies including *in situ* product removal (ISPR) to maintain sub-threshold concentrations of 7-Br-Trp as has been shown for l-phenylalanine separation and concentration by reactive-extraction with liquid-liquid centrifuges in a fed-batch fermentation process with recombinant *E. coli* (Rüffer et al., 2004).

Halogenated amino acids such as 7-Br-Trp are relevant for peptide synthesis, since they can be converted further by Pd-catalyzed cross coupling and nucleophilic substitution reactions (Diederich and Stang, 2008). Various halogenated forms of tryptophan and its derivatives may have potential in the synthesis of serotonin and melatonin agonists or antagonists (Frese et al., 2014). As shown here, based on the insight from enzyme catalysis using pure RebH, crude RebH preparations or CLEAs containing RebH, a fermentative process based on RebH was developed and adjusted to yield either 7-Cl-Trp or 7-Br-Trp by *C. glutamicum in vivo*. Since halogenases such as RebH, PrnA, or BrvH differ in their substrate spectra and regioselectivities, the fermentative approach holds the potential to be extended for various halogenation processes starting from glucose and halide salts *in vivo* provided that the halogenated products do not interfere with vital cellular functions and can be exported out of the cell efficiently.

## Data Availability

This manuscript contains previously unpublished data. All data sets generated for this study are included in the manuscript and the supplementary files.

## Author Contributions

KV and SD carried out experimental procedures of the present study. KV, SD, JR, J-HL, NS, and VW analyzed data. KV prepared a draft of the manuscript. KV, J-HL, and VW finalized the manuscript. VW coordinated the study. All authors read and approved the final version of the manuscript.

### Conflict of Interest Statement

The authors declare that the research was conducted in the absence of any commercial or financial relationships that could be construed as a potential conflict of interest.
